# Safety and efficacy of thoracoscopic wedge resection for elderly high-risk patients with stage I peripheral non-small-cell lung cancer

**DOI:** 10.1186/1749-8090-8-231

**Published:** 2013-12-21

**Authors:** Ling Lin, Dingzhong Hu, Chenxi Zhong, Heng Zhao

**Affiliations:** 1 Department of Thoracic Surgery, Shanghai Chest Hospital, School of Medicine, Shanghai Jiaotong University, 241 West Huaihai Road, Shanghai 200030, China

**Keywords:** Elderly, Early lung cancer, Thoracoscope

## Abstract

**Background:**

Elderly patients with severe cardiopulmonary and other system dysfunctions are unable to tolerate pulmonary lobectomy. This study aimed to evaluate the risk and efficacy of wedge resection under video-assisted thoracoscopic surgery (VATS) on elderly high-risk patients with stage I peripheral non-small-cell lung cancer (PNSCLC).

**Methods:**

Elderly patients (≥70 years) with suspected PNSCLC were divided into high-risk group and conventional risk group. The high-risk patients confirmed in stage I by the examination of positron emission tomography computed tomography (PET-CT) and the postoperative patients in stage I PNSCLC with negative incisal margin were treated with VATS wedge resection. The conventional risk patients were treated with VATS radical resection and systematic lymphadenectomy. The clinical and pathological data were recorded. The total survival, tumor-free survival, recurrence time and style of patients were followed up.

**Results:**

The operative time and blood loss of the VATS wedge resection group (69.4 ± 15.5 min, 52.1 ± 11.2 ml) were significantly less than those of the VATS radical resection group (128 ± 35.5 min, 217.9 ± 87.1 ml). Neither groups had postoperative death. The overall and tumor-free survival rate of the VATS wedge resection group within three years were 66.7% and 60.0%, and those of the VATS radical resection group were 93.8% and 94.1%, without significant difference (*P* > 0.05). The recurrence rates of the VATS wedge resection group and VATS radical resection group were 14.3% and 3.0%, without significant difference (*P* > 0.05).

**Conclusion:**

It is safe, minimally invasive and meaningful to perform VATS wedge resection on the elderly high-risk patients with stage I PNSCLC.

## Background

Lung cancer is a leading cause of cancer-related mortality and a common disease of the elderly. The statistics of National Cancer Institute have shown that the lung cancer mainly occurs in those aged between 75 and 79 and the peak mortality rate is between 75 and 84 years, depending on gender [1]. The prognosis of lung cancer is poor and less than 15% of patients could survive 5 years after diagnosis. The lack of efficient diagnostic methods and successful treatment for early detection and metastatic disease caused the poor prognosis [2].

Approximately 85% of all lung cancers are classified as non-small-cell lung cancer (NSCLC), 10% are small cell lung cancer and 5% other histological variants with distinct biological behavior, genetic alterations, and therapy [3,4]. The NSCLC is potentially curable by surgical resection if it is discovered at an early stage [5]. And the locally advanced NSCLC is treated with radiotherapy or chemotherapy [6]. Pulmonary lobectomy is the removal of one of the five lobes of the lung and wedge resection is the removal of the lung tumor and a rim of healthy lung tissue around the tumor [1,7]. Pulmonary lobectomy combined with systematic lymphadenectomy or sampling is the accepted standard treatment for lung cancer currently. However, the risk of operation is gradually rising along with the rise of age [8]. Some clinical, socioeconomic, and surgeon factors were statistically significantly associated with the choice of surgical resection for early-stage NSCLC [9].

The technique of video-assisted thoracic surgery (VATS) is a well-established technique in the armamentarium of the thoracic surgeon and currently indicated for a wide spectrum of pulmonary diseases, including primary lung cancer [10,11]. The risk of pulmonary lobectomy for elderly patients is greatly reduced along with the maturity of thoracoscopic lobectomy and lymphadenectomy , and advanced age is no longer a barrier of the VATS radical resection [12]. Nevertheless, a lot of elderly patients usually have severe cardiopulmonary and other system dysfunctions and are unable to tolerate the pulmonary lobectomy. It could only be a local excision though these elderly high-risk patients treated with VATS. And the systematic lymphadenectomy or sampling might be simplified or omitted because of the operative risk. Thus, patients who severely compromised pulmonary function, advanced age or other extensive comorbidity cannot tolerate a full lobectomy, a more limited operation is recommended [13-15].

Therefore, we undertook this study to evaluate the operative risk of VATS wedge resection performed on elderly high-risk patients with stage I peripheral non-small-cell lung cancer (PNSCLC) and the therapeutic effects of the VATS wedge resection for these patients.

## Methods

### Studying subjects

All of the patients 70 or above 70 years old diagnosed from May 2008 to June 2012 with suspected PNSCLC were collected. This study was approved by the Research Ethics Committee of Shanghai Jiao Tong University School of Medicine and all participants at the Shanghai Chest Hospital gave written informed consent.

Physical examination and routine examination of these patients were performed. Routine check-up included the examinations of preoperative blood biochemical indexes, chest radiography, chest CT, electrocardiogram, heart of color Doppler ultrasound, lung function and arterial blood gases.

The patients were identified to be high-risk if one of the following descriptions had conformed to the medical history and preliminary results. Otherwise, the patients were subjected into the conventional risk group. The criteria for high-risk were shown as follows:

(1) Pulmonary function test: the percentage ratio of forced expiratory volume in 1 second (FEV1) to predicted value < 60% or the absolute value of FEV1 < 1.2 L or the percentage ratio of carbon monoxide diffusion capacity (DLCO) to predicted value < 60%. (2) Arterial blood gas analysis of suction air in a calm case: PaO_2_ ≤ 60 mmHg or PaCO_2_ ≥50 mmHg. (3) Including three items of the following symptoms: Primary hypertension in or above stage II, diabetes for more than 2 years and abnormal levels of glycated hemoglobin and microalbuminuria with poor glucose control, undergoing coronary stenting of myocardial infarction or coronary artery within two years, moderate and increased level of valvular disease, frequent ventricular arrhythmia, the left ventricular ejection fraction < 60%, a history of cerebrovascular accident within two years, undergoing pulmonary lobectomy and more serious operations, renal insufficiency and the value of serum creatinine to the normal value ≥ 2, compensated cirrhosis, notable marasmus with reduced total protein, albumin and prealbumin of blood, a history of hemorrhagic disease with abnormal clotting.

The examinations of positron emission tomography computed tomography (PET-CT) for patients considered to be high-risk were performed. The patients with stage I PNSCLC received the treatment of VATS lung wedge resection when the results of PET-CT had indicated that the lung vicinity tubercle was likely to be malignant. The case would be counted into the elderly high-risk group with the treatment of VAST wedge resection if the patient was confirmed to be in stage I by postoperative pathology and had a negative surgical margin.

The preoperative patients with stage I PNSCLC of conventional risk group were received the VAST radical resection and the systematic lymphadenectomy. The case would be counted into the elderly conventional risk group with the treatment of VAST radical resection if the patient was confirmed to be in the stage I by postoperative pathology.

By the end of June 2012, the cases of the elderly high-risk group with the treatment of VAST wedge resection and the elderly conventional risk group with the treatment of VAST radical resection were 14 and 33, respectively.

### Operative procedure

The VAST radical resection group: The patients were required with conventional lateral position. A 1.5 cm incision was made in the 8th rib under scapula as the exits and entrances of cut staplers and sponge clamp. A 4-5 cm incision was made at inner side of the anterior axillary line between the 4th and 5th rib acting as the operation recesses and the outlet of samples according to the resected pulmonary lobe. Bronchia, pulmonary vasculature and inter-lobar fissure were cut off or sutured by the corresponding endoscopic cut stapler respectively. Then systematic lymphadenectomy was performed. The VAST wedge resection group: The operative incision was the same as the previous group or the incision in the 8th rib under scapula was omitted, and the patients were required with conventional lateral position. Wedge-shaped excision of lung was performed 2 cm from the edge of the tumor using endoscopic cut stapler after the location of the tumor was determinated by palpation and video.

The frozen sections of postoperative specimens were prepared immediately. The lymphadenectomy or sampling wasn’t performed on the patients of the wedge resection group.

### Data collecting

The age, gender, site of the tumor, operative time, blood loss, postoperative complications, hospital stay, postoperative deaths within 30 days, size of the tumor, pleural invasion, T stage and pathological stage of the 47 cases were collected. And the histological type and differentiation of tumor were determinated by histological sections. Postoperative complications were the symptoms that occurred within 30 days post operation including myocardial infarction, arrhythmia, pulmonary infarction, lower limb venous embolism, postoperative leakage above 7 days, rebleeding, bronchopleural fistula, chest or lung infection, wound infection, chylothorax and respiratory failure.

The overall survival, tumor-free survival, time and patterns of recurrence of all patients were collected by follow-up visits. It was necessary to provide pathological evidence of the recurrence and metastasis of tumor as much as possible in principle, such as, lymph node biopsies or needle biopsy of lung. It could be inferred as recurrence or metastasis when more than two different imaging examinations had demonstrated the possibility of metastasis in the condition that unable to obtain pathology.

### Statistical analysis

Statistical analysis was performed by the SPSS version 12.0 statistical software. The measurement data were expressed as $\bar{X}\pm \mathrm{SD}$ and analyzed by *T*-test. Meanwhile, *χ*^2^-test was used to analyze the enumeration data. The method of Kaplan-Meier was applied to analyze the overall survival and the tumor-free survival, and the statistical evaluation was conducted by Log-Rank test. There is significantly statistical difference when *P* <0.05.

## Results

### Comparison of the clinical and pathological data

The clinical and pathological data were shown in Table 1. There was no significant difference in the age, gender, position of the tumor, tumor size, histological type, differentiation, pleural invasion, T stage and pathological stage between the two groups. The histological types of VATS wedge resection group were 2 cases of bronchioloalveolar carcinoma, 9 cases of adenomatous carcinoma, 2 cases of squamous carcinoma and 1 case of adenosquamous carcinoma. The histological types of VATS radical resection group were 1 case of bronchioloalveolar carcinoma, 23 cases of adenomatous carcinoma, 5 cases of squamous carcinoma, 1 case of adenosquamous carcinoma and 3 cases of large cell carcinoma. Though the clinical and pathological data of the two groups had no obvious difference, the proportions of bronchioloalveolar carcinoma and high differentiated tumor in the VATS wedge resection group were slightly increased.

**Table 1:** Comparison of the clinical and pathological data

		**VATS wedge resection (n = 14)**	**VATS radical resection (n = 33)**	***P *** **value**
Age (years)		74.93 ± 3.12	72.82 ± 2.92	0.943
Gender	Male	9	20	0.812
	Female	5	13	
Site of the tumor	Right upper lobe	5 (35.7%)	9 (27.3%)	0.721
	Right middle lobe	1 (7.1%)	4 (12.1%)	
	Right lower lobe	1 (7.1%)	7 (21.2%)	
	Left upper lobe	5 (35.7%)	8 (24.2%)	
	Left lower lobe	2 (14.3%)	5 (15.2%)	
Histological type	Bronchioloalveolar carcinoma	2 (14.3%)	1 (3.0%)	0.527
	Adenomatous carcinoma	9 (64.3%)	23 (69.7%)	
	Squamous carcinoma	2 (14.3%)	5 (15.2%)	
	Others	1 (7.1%)	4 (12.1%)	
Tumor size (cm)		2.24 ± 0.56	2.33 ± 0.82	0.094
Differentiation	Low	1 (7.1%)	8 (24.2%)	0.134
	Medium	9 (64.3%)	22 (66.7%)	
	High	4 (28.6%)	3 (9.1%)	
Pleural invasion	With	4 (28.6%)	8 (24.2%)	0.756
	Without	10 (71.4%)	25 (75.8%)	
T-stage	1a	6 (42.9%)	12 (36.4%)	0.865
	1b	4 (28.6%)	9 (27.3%)	
	2a	4 (28.6%)	12 (36.4%)	
Pathological stage	IA	10 (71.4%)	21 (63.6%)	0.606

### Comparison of the postoperative recovery and follow-up visits

The postoperative recovery and follow-up visits of the two groups were shown in Table 2. There was no significant difference in hospital stay, average follow-up time and postoperative complications between the two groups. Both of the two groups had no postoperative death within 30 days. The operative time of VATS wedge resection group was 69.4 ± 15.5 min, and was significantly reduced compared with the 128 ± 35.5 min of VATS radical resection group (*P* < 0.05). The blood loss of VATS wedge resection group was 52.1 ± 11.2 ml, and was significantly reduced compared with the 217.9 ± 87.1 ml of VATS radical resection group (*P* < 0.05).

**Table 2:** Comparison of the postoperative recovery and follow-up visits

	**VATS wedge resection (n = 14)**	**VATS radical resection (n = 33)**	***P *** **value**
Operative time (min)	69.4 ± 15.5	128 ± 35.5	0.020
Blood loss (ml)	52.1 ± 11.2	217.9 ± 87.1	0.025
Postoperative complications	3 (21.4%)	4 (12.2%)	0.412
Hospital stay (days)	8.14 ± 2.62	8.67 ± 2.18	0.449
Postoperative deaths within 30 days	0	0	—
follow-up time (months)	17.93 ± 16.14	20.85 ± 11.71	0.094
Postoperative recurrence	2 (14.3%)	1 (3.0%)	0.149
Local recurrence	1 (7.1%)	0 (0%)	0.121
Distant metastasis	1 (7.1%)	1 (3.0%)	0.523

The three cases of the complications of the VATS wedge resection group were atrial fibrillation, pneumonia and air leakage above 7 days. The four cases of the VATS radical resection group were two cases of atrial fibrillation, one case of pneumonia and one case of air leakage above 7 days.

The recurrence rates of the VATS wedge resection group and VATS radical resection group were 14.3% and 3.0%, without significant difference (*P* > 0.05). The two cases of the postoperative recurrence of the VATS wedge resection group were local recurrence and distant metastasis according to the follow-up visit. The case of local recurrence was a 79-year-old patient whose histological type was bronchioloalveolar carcinoma. The recurrence of the resection occurred at 26 months after the operation and the patient died of severe lung infections at 32 months postoperatively. The case of distant metastasis was a 78-year-old patient whose histological type was moderately differentiated adenocarcinoma. The brain metastase was appeared at 22 months postoperatively and the bone metastases occurred after the γ-knife treatment, and the patient died of lung cancer at 42 months after the operation. There was only one distant metastasis of the VATS radical resection group according to the follow-up visit. It was a 74-year-old patient with large cell carcinoma. A wide range of bone metastases and brain metastases were appeared at 28 months postoperatively and the patient died of lung cancer at 21 months postoperatively. There was not any death caused by anything other than lung cancer during this study period.

**Figure 1:**
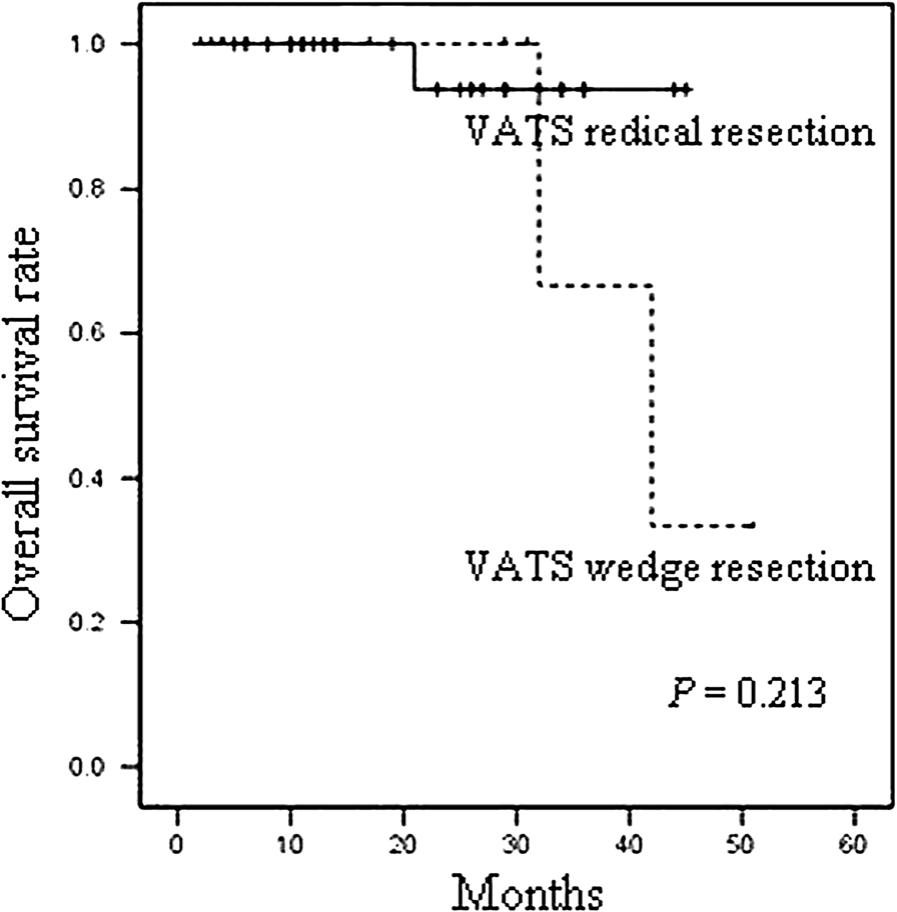
The overall survival of VATS radical resection group and VATS wedge resection group.

**Figure 2:**
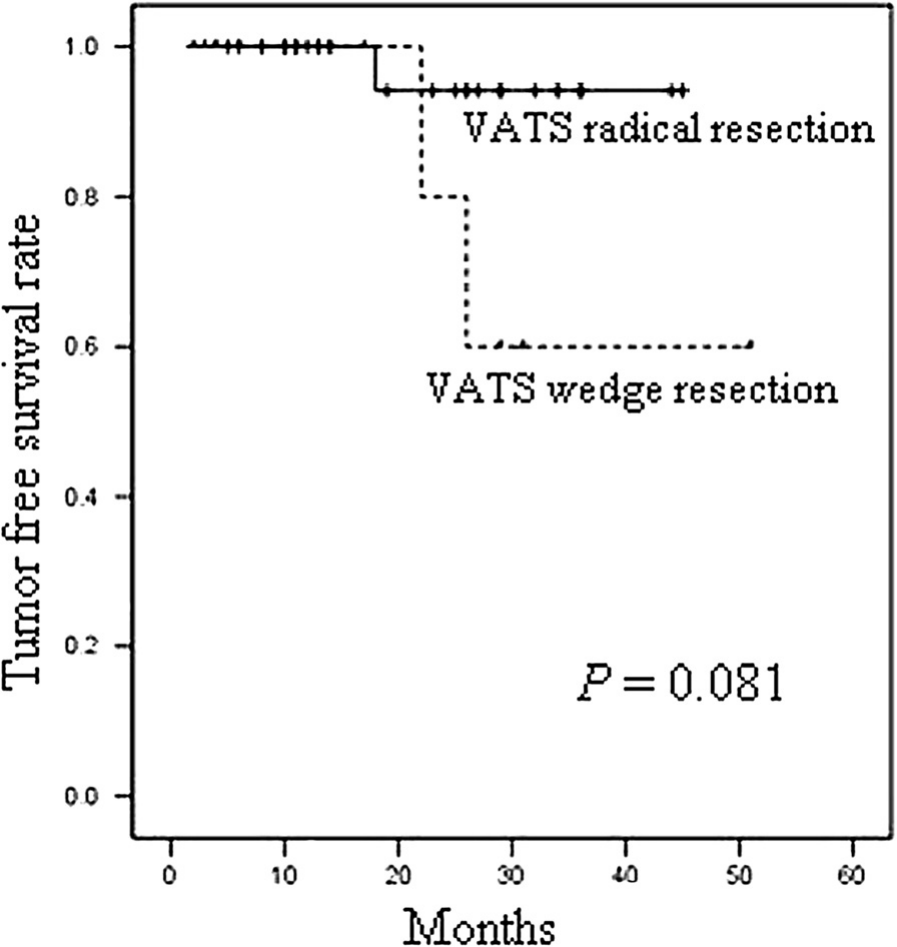
The tumor-free survival of VATS radical resection group and VATS wedge resection group.

As shown in Figure 1 and Figure 2, the statistical results of survival indicated that the overall survival rate and tumor-free survival rate of the VATS wedge resection group within three years were 66.7% and 60.0%. And the overall survival rate and tumor-free survival rate of the VATS radical resection group within three years were 93.8% and 94.1%. There was no statistically significant difference in the overall survival rate and tumor-free survival rate between the two groups. And the *P* values of the two groups were 0.213 and 0.081, respectively.

## Discussion

Along with the aging of the society and the universalness of the physical examination, there are more and more elderly patients with stage I lung cancer. In this study, there were 8 elderly high-risk cases from May 2008 to December 2011 while the number of elderly high-risk cases was 6 in the first half of 2012. Therefore, the number of elderly high-risk cases is increasing and this situation is becoming a trend.

There is significant relationship between the rate of postoperative complications and the rate of preoperative complications [16]. So, our determination to perform the VATS radical resection is usually disturbed by the preoperative complications of patients. How to choose a proper operation method according to the specific situation of the elderly patients has not been determined. Meanwhile, the balance between survival and risk of the operation is the key to whole choice.

The safety of the VATS wedge resection performed on elderly high-risk patients was the primary issue to be considered in this study, and a standard for the risk assessment was established according to the related literatures and our previous experience in operation [17-19]. It has been reported that wedge resections could obtain a disease-free and overall survival equivalent to lobectomies and segmentectomies in stage IA NSCLC patients [20]. However, the prognostic impact of wedge resections and segmentectomies is still under debate. Wedge resections with lymph node sampling can be more easily performed using VATS techniques than segmentectomy. In this study, the elderly patients with stage I PNSCLC were divided into two groups: the high-risk group and conventional risk group. The VATS wedge resection was conducted on the patients of high-risk group because the risk of VATS radical resection for high-risk group was extremely high. Meanwhile, the group of conventional risk received the treatment of VATS radical resection. Both of the two groups had no postoperative death, and the incidence of complications was 12.2% in the conventional risk group. It has been reported that the incidence of complications in patients over 80 years old received the treatment of VATS radical resection is 18-35% [21-23]. The incidence of complications of conventional risk group was lower than the reports presented, which may be related to the age of the conventional risk group in our study. There was only one case of patient above 80 years and the ages of the conventional risk group were mainly between 70-79 years. The incidence of complications of high-risk group was 21.4% and had no obvious difference compared with the conventional risk group. The operative time and blood loss of high-risk group were lower than the conventional risk group since the VATS wedge resection was conducted on the patients of high-risk group. Therefore, this difference was caused by the options of operation. Long operative time and more bleeding are the dangerous factors that lead to the high incidence of complications and mortality [16,24]. Thus, it is feasible and safe to perform VATS wedge resection on the elderly high-risk patients.

Another critical factor that affected our decision was survival since the operative risk of performing VATS wedge resection on the elderly high-risk patients was acceptable. Vrdoljal et al reported that the average survival time was 17 months and the survival rate was 20% within two years while patients with stage I lung cancer had no treatment [25]. Morita et al analyzed the therapeutic effects of 149 patients with stage I PNSCLC that received the radical radiotherapy. The analysis results showed that the average survival time was 27.2 months and the survival rates were 34.2% and 22.2% within 3 and 5 years respectively [26]. The related foreign literatures have reported that the survival rates were 76-84.2% and 55.6-75% within 3 and 5 years after the patients above 80 years with stage I lung cancer received the treatment of VATS radical resection [16,22,23,27]. In this study, the total survival rate and tumor-free survival rate of the elderly high-risk group treated with the VATS wedge resection within 3 years were 66.7% and 60.0%, and those of the conventional risk group treated with the VATS radical resection and systematic lymphadenectomy within 3 years were 93.8% and 94.1%. Thus, the total survival rate of conventional risk group was higher than the reports presented above. The reason was that the patients of the conventional risk group were 10 years younger and had no obvious contraindications and fewer complications. The patients of the elderly high-risk group in this study were usually considered to be unable to tolerate the operation in the past. The survival rate of the elderly high-risk group within 3 years after the VATS wedge resection was significantly higher than the reported patients with no treatment or treated with radical radiotherapy. The research about randomized controlled trial of the early lung cancer by Lung Cancer Study Group (LCSG) in 1995 showed that there was no significant difference in survival rate within 3 years between the pulmonary lobectomy and local resection [28]. Large sample retrospective analysis by Mery et al in 2005 showed the effect of local resection on the total survival rate depending on age and there was no significant difference in survival rate between the local resection and pulmonary lobectomy for the patients above 71 years [1]. The research result announced in Japanese multicenter by Okada et al in 2006 showed that there was no significant difference in recurrence rate between local resection and pulmonary lobectomy [20]. In this study, there was no significant difference in survival rate, local recurrence, distant metastasis and total recurrence rate between the two groups. Thus, these results were similar with the conclusion of randomized study and large sample retrospective analysis aboard.

Based on the above research results, it is reasonable to believe that the prognosis of elderly high-risk patients with stage I lung cancer that are treated with VATS wedge resection will be much better than the patients that receive no treatment. What’s more, the medium-term survival rate and relapse metastasis rate of the VATS wedge resection will not be worse than the VATS radical resection and the survival benefit of the VATS wedge resection is very obvious. However, there are some weaknesses and limitations in our study, such as the small sample size and short-term follow-up. Therefore, further researches with larger sample size and longer follow-up are needed to evaluate the safety and efficacy of thoracoscopic wedge resection for elderly high-risk patients with stage I peripheral non-small-cell lung cancer.

## Conclusions

In conclusion, it appears to be safe and meaningful for tumorous therapy when the elderly high-risk patients with stage I PNSCLC were treated with VATS wedge resection, and the VATS wedge resection could avoid the problem that patients died of lung cancer in early stage. What’s more, the comparison of long-term effect still needs follow-up visits for longer time and more patients in consideration of the limitation of clinical time.

## Abbreviations

VATS: Video-assisted thoracoscopic surgery; PNSCLC: Peripheral non-small-cell lung cancer; PET-CT: Positron emission tomography computed tomography; FEV1: Forced expiratory volume in 1 second; DLCO: Carbon monoxide diffusion capacity; LCSG: Lung cancer study group.

## Competing interests

The authors declare that they have no competing interest.

## Authors’ contributions

Design the experiments: LL, DZH. Do and interpret the experiments: LL, CXZ, HZ. Write the manuscript: LL. All authors read and approved the final manuscript. Thanks very much for your efforts and vable time.
